# A longitudinal population-based analysis of relationship status and mortality in KwaZulu-Natal, South Africa 2001–2011

**DOI:** 10.1136/jech-2014-205408

**Published:** 2015-08-07

**Authors:** Melanie Channon, Victoria Hosegood, Nuala McGrath

**Affiliations:** 1 Oxford Institute of Population Ageing, University of Oxford, Oxford, UK; 2 Department of Social Statistics and Demography, University of Southampton, Southampton, UK; 3 Africa Centre for Health and Population Studies, University of KwaZulu-Natal, Somkhele, South Africa; 4 Department of Social Statistics and Demography and Academic Unit of Primary Care and Population Sciences, University of Southampton, Southampton, UK

**Keywords:** MORTALITY, MARITAL STATUS, HIV

## Abstract

**Background:**

Mortality risk is lower in married than in unmarried men and women. However, little is known about the association between mortality and relationship status in South Africa where marriage rates are low, migration is common, many couples are not co-resident and HIV prevalence is high.

**Method:**

Using demographic surveillance data collected from 2001 to 2011, relationship status was categorised as conjugal (partners belong to the same household), non-conjugal (partners do not belong to the same household) or not partnered. Rates of relationship formation and dissolution were calculated by age and sex. Controlling for antiretroviral treatment (ART) introduction in 2005 as well as education, sex-specific and age-specific Cox proportional hazards models were used to investigate the association between relationship status and (1) all-cause mortality and (2) non-AIDS mortality.

**Results:**

Before 2005, individuals in conjugal relationships had a lower hazard of all-cause mortality in all age groups than not partnered men and women. Non-conjugal relationships lowered the risk of dying compared with not partnered men and women in fewer age groups. After ART introduction, the protective association of conjugal relationships was weaker but remained generally significant for men and women but not in non-conjugal relationships. In the later period, the association is reversed in young men (20–29 years) with mortality higher in conjugal and non-conjugal relationships compared with men not partnered. The analysis of non-AIDS deaths provided similar results.

**Conclusions:**

The higher degree of social connections within a shared household environment that characterises conjugal relationships affords men and women greater protection against mortality.

## Background

It is well documented that in many contexts marital status has a strong association with mortality.^1–4^ It has generally been found that being married is associated with a lower mortality rate and that in many cases the association is stronger for males.^5–7^ It has also been found that the protective effect of marriage is weaker at older ages.^8^ ^9^ However, with a few exceptions, little attempt has been made to study the effect of relationship status as a time-varying covariate; instead, marital status has typically been treated as fixed at a certain point in time,^1^ ^7^ ^10^ and very few studies consider the association between different types of relationship status (eg, non-marital) and mortality. Furthermore, we could not find any studies on the association between mortality and marital status (or social relationships more generally) in the African context as the majority of studies have been conducted in North America or Western Europe.^3^ ^4^

We use longitudinal, population-based data available in the Africa Centre Demographic Information System (ACDIS) to explore whether the protective association between relationship status and mortality holds in a rural South African context, and to extend our understanding of relationship formation and dissolution in a rural South African population.

The level and patterns of HIV incidence and prevalence, HIV treatment and mortality have been well described in this population.^11–13^ In South Africa, marriage among some communities has been declining for decades, particularly in KwaZulu-Natal, where, by 2008/2009, 12% of women aged 20–45 years were currently married and fewer than one-third of women aged 45–49 years were ever married.^14–16^ Non-marital relationships are very common but cohabitation is not as common as in other settings and a variety of different residential arrangements exist between both marital and non-marital partners.^14^ Thus, in this setting, we argue that there is a need to define relationships in a different manner to the standard married/non-married dichotomy. We use the concept of a conjugal relationship, which is considered to be a sexual relationship where both partners are members of the same household (whether resident or non-resident), and thus this reflects a high level of social recognition.

In this paper, we estimate formation and dissolution rates for men and women's relationships from 2001 to 2011 in KwaZulu-Natal and compare the risk of mortality for people in a conjugal relationship, a non-conjugal relationship and those not partnered by sex in the periods before and after HIV antiretroviral treatment (ART) was available.

## Data and methods

### Data

The data used in this paper were collected as part of the ACDIS, an ongoing demographic surveillance system (DSS) in KwaZulu-Natal.^17^ The ACDIS collects information on all 89 000 members of households within the site; however, individuals can have multiple household memberships and/or be a member of a household without being resident in the same geographic location as the household.^18^ The reason for this is that defining households only in the sense of co-residence ignores the dynamic and complex nature of households in this area; non-resident members can have relationships with other household members (eg, spouse, parent, child), and the health and well-being of non-resident members may be closely related to the health and welfare of resident members and vice versa, and in the South African context non-resident adults make frequent return visits.^18^ ^19^ Demographic data on members of the DSS are updated through a routine household visit which varied in frequency over time between two and three times a year.^17^

At each routine household visit, the current relationship status of all individuals is recorded. However, the relationship status does not indicate who the partner is or when the relationship started. When the two partners are members of the same household, the couple are classified as a conjugal relationship and additional information is collected including the identity of the partner, the start date of the relationship, the date of customary or civil marriage (if any) and the end date (if applicable).

Given that relationship status does not remain constant over time and our analysis was conducted over a period of more than a decade, we considered relationship status as time-varying in all our models. By considering relationship status as time-varying, we are specifically studying the association between mortality risk and *current* relationship status, whereas previous research has generally operationalised relationship (or marital) status for a fixed time point. Over long periods, treating relationship status as static would potentially lead to misleading results. At any point in time, there are three possible mutually exclusive relationship states: not partnered, in a non-conjugal relationship (ie, partnered but not linked in the data and not members of the same household) and in a conjugal relationship.

### Methods

All adults aged 20 years and older on 1 January 2001 were followed up until death, the ending of any membership of a household resident in the study area, or 1 December 2011 when they were censored. Person-time in a conjugal relationship was calculated using the documented start and end dates of the conjugal relationship. For person-time in a non-conjugal relationship, it was assumed that time in this state started on the date his or her non-conjugal relationship was first reported during a routine household visit. A non-conjugal relationship was assumed to have ended on the date the routine household visit that documented the person's relationship status had reverted to unpartnered.

**Figure 1:**
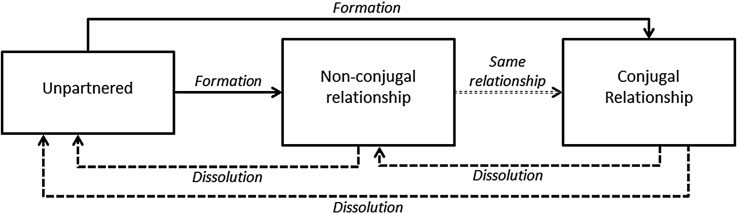
Illustration of possible transitions between relationship states.

Relationship formation and dissolution rates were calculated as the number of formations or dissolutions divided by the number of person-years experienced at risk of that event presented per 1000 person-years. The transitions between the three relationship states are summarised in figure 1. A relationship formation was defined to be a change from not being in a relationship to being in either a non-conjugal or a conjugal relationship. Changes from non-conjugal to conjugal relationships are assumed to be a change in stage of relationship, rather than a new relationship. Individuals in the denominator were not partnered, that is, at risk of an event. Dissolution is defined as a change from being in a non-conjugal or conjugal relationship to not being partnered through separation, divorce or widowhood. Person-time at risk of dissolution was calculated as the sum of all periods where an individual was in a relationship (conjugal or non-conjugal).

In order to model the association between relationship status and mortality, we used Cox proportional hazards models.^20^ The proportional hazards assumption was tested using Schoenfeld residuals.^21^ Relationship status was included in the models as a time-varying covariate. All of the multivariate analyses controlled for age (using linear and quadratic terms) and highest reported educational attainment (as a categorical variable). Educational attainment was split into five categories: <1 year, primary school, secondary (not matriculated), matriculated or higher and unknown/missing. Public roll-out of HIV treatment began in the study area in November 2004^22^ following which adult mortality declined.^11^ Therefore, we examine the association between relationship status and mortality before and after 2005 using time-varying coefficients.^23^

We also repeated the analyses for non-AIDS deaths only where individuals were censored at the time of their AIDS death (a competing risk analysis). The rationale for focusing on non-AIDS deaths is to provide a direct comparison with previous studies on marital status and mortality in low general HIV prevalence contexts. Furthermore, HIV and ART status are not available for both partners in most cases and it would not be possible to appropriately adjust for confounding by the timing of HIV status and ART initiation within couples. Previous studies have found mixed results with marriage associated with both increased^24^ ^25^ and decreased^26^ HIV risk.

## Results

In January 2001, 32% of women were not partnered (table 1), 42% were in a non-conjugal relationship and 26% were in a conjugal relationship. However, this hides a great deal of variation by age, with 70% of those aged 20–29 years in a non-conjugal relationship compared with <10% of those aged 60 and over. Those aged 60 and over were particularly likely to be not partnered, while those aged 50–59 years were most likely to be in a conjugal relationship.

**Table 1:** Distribution of relationship status on 1 January 2001 in the ACDIS, by age and sex

	20–29 Years	30–39 Years	40–49 Years	50–59 Years	60+ Years	Total
**All**						
Unpartnered						
n	3807	1051	928	933	2209	8928
%	25.3	10.6	14.3	25.1	47.1	22.4
In a non-conjugal relationship						
n	9779	4878	1763	548	415	17 383
%	64.9	49.3	27.1	14.8	8.8	43.6
In a conjugal relationship						
n	1483	3958	3811	2234	2067	13 553
%	9.8	40.0	58.6	60.1	44.1	34.0
Among conjugal relationships: currently married						
%	18.5	*49*.*1*	*63*.*2*	*72*.*9*	*79*.*8*	*58*.*3*
Among conjugal relationships: cohabiting in the DSA						
%	*57*.*9*	*60*.*5*	*61*.*4*	*65*.*0*	*78*.*1*	*63*.*9*
Total						
n	15 069	9887	6502	3715	4691	39 864
%	100	100	100	100	100	100

**Females**						
Unpartnered						
n	1312	574	673	778	2013	5350
%	16.6	10.7	19.3	37.2	65.6	32.3
In a non-conjugal relationship						
n	5551	2542	966	295	250	9604
%	70.0	47.5	27.8	14.1	8.1	41.5
In a conjugal relationship						
n	1065	2236	1842	1019	808	6970
%	13.4	41.8	52.9	48.7	26.3	26.2
Among conjugal relationships: currently married						
%	*22*.*3*	*54*.*0*	*66*.*1*	*78*.*9*	*83*.*5*	*59*.*4*
Among conjugal relationships: cohabiting in the DSA						
%	*58*.*0*	*55*.*8*	*54*.*5*	*64*.*2*	*74*.*8*	*59*.*2*
Total						
n	7928	5352	3481	2092	3071	21 924
%	100	100	100	100	100	100

**Males**						
Unpartnered						
n	2495	477	255	155	196	3578
%	34.9	10.5	8.4	9.6	12.1	19.9
In a non-conjugal relationship						
n	4228	2336	797	253	165	7779
%	59.2	51.5	26.4	15.6	10.2	43.4
In a conjugal relationship						
n	418	1722	1969	1215	1259	6583
%	5.9	38.0	65.2	74.9	77.7	36.7
Among conjugal relationships: currently married						
%	*8*.*9*	*42*.*8*	*60*.*4*	*67*.*8*	*77*.*4*	*57*.*2*
Among conjugal relationships: cohabiting in the DSA						
%	*57*.*8*	*66*.*7*	*67*.*8*	*65*.*7*	*80*.*2*	*68*.*9*
Total						
n	7141	4535	3021	1623	1620	17 940
%	100	100	100	100	100	100

ACDIS, Africa Centre Demographic Information System; DSA, Demographic Surveillance Area.

Men were generally more likely to be in a conjugal relationship than women, especially at older ages. This is the consequence of women's longer survival and their partners dying before them, especially since in more than 85% of conjugal relationships the woman was younger than the man with a mean age difference of more than 6 years. Men were much less likely than women to be in a relationship in the 20–29 year age group, but at older ages men were more likely to be in a relationship. Men aged 20–24 years were especially unlikely to be in any form of relationship, with only 2% in a conjugal relationship and 45% not partnered. Two-thirds of men aged 25–29 years were in a relationship; however, these relationships were predominantly non-conjugal.

Overall, 64% of couples in a conjugal relationship on 1 January 2001 were cohabiting and 58% were married. For older couples in a conjugal relationship, cohabiting was more common (78% among those aged over 60 years), while for younger couples the most common reason for not cohabiting was that the male was outside the DSS. Marriage also varied by age with around 80% of conjugal couples being married in the 60 and over age group, but less than a fifth in the age group of 20–29 years.

**Figure 2:**
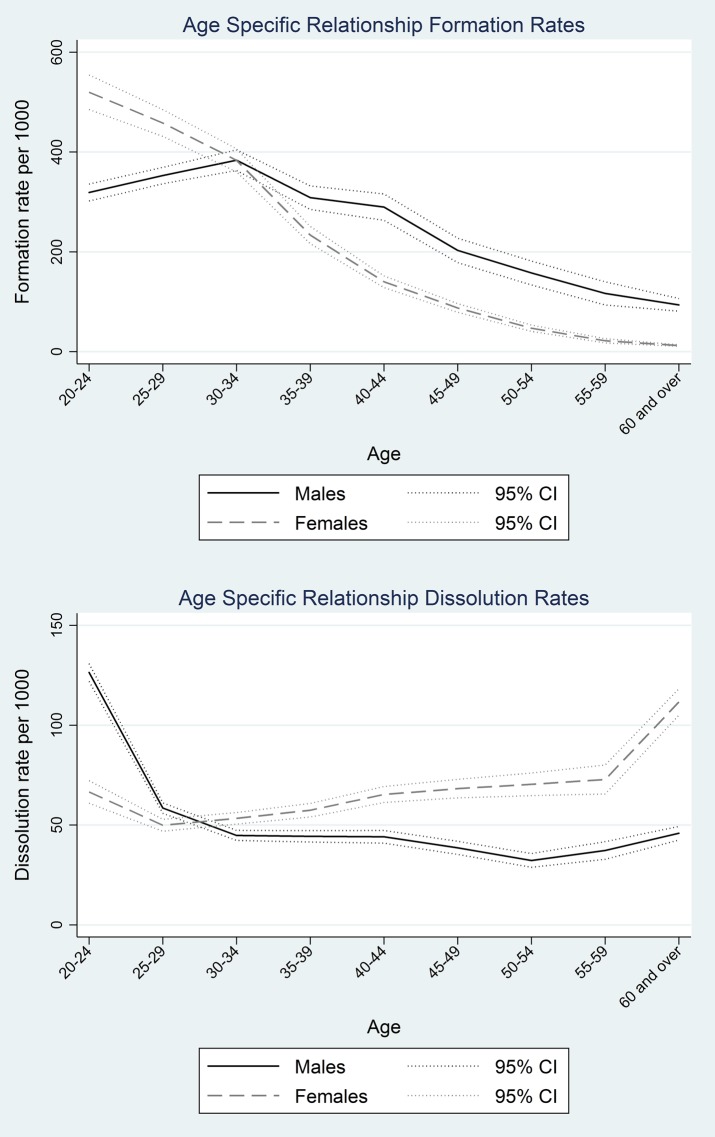
Age-specific relationship formation and dissolution rates, by sex.

Table 2 shows age-specific formation and dissolution rates of relationships. Given the sex differentials in relationship status by age highlighted in table 1, sex-specific formation and dissolution rates were calculated. Figure 2 shows point estimates for these rates with 95% CIs. Formation rates were highest for females aged 20–29 years and males aged 30–39 years. For both sexes, formation rates declined substantially in the older age groups, although for those aged 60 years and older relationship formation was much more common for males than for females. Indeed, for those aged 60 years and older, the relationship formation rates for males and females were 93 and 12/1000 person-years, respectively, and this difference was statistically significant.

**Table 2:** Relationship formation and dissolution patterns by age and sex in the Africa Centre Demographic Information System, 2001–2011

	Females			Males		
Age (years)	Person-years at risk	Per cent with at least one formation	Rate per 1000	Person-years at risk	Per cent with at least one formation	Rate per 1000
Formations						
20–29	4109	67.2	483	9294	63.2	337
30–39	5828	50.5	305	5610	57.7	355
40–49	8471	28.6	112	2908	45.8	251
50–59	9026	10.8	34	1884	30.4	140
60+	22 783	6.6	12	2258	25.0	93
Dissolutions						
20–29	29 639	17.1	54	27 958	18.6	68
30–39	43 313	18.4	55	48 362	14.5	43
40–49	27 885	23.1	67	31 076	13.1	38
50–59	13 767	24.1	71	18 093	11.3	32
60+	9791	44.4	112	15 623	17.3	41

Dissolution rates peaked for men in the youngest age group and women in the oldest age group. The high dissolution rates seen at either end of the age range are most probably related to different processes. Dissolutions in the younger age group are associated with relationship instability, whereas for women at older ages dissolution is primarily due to the death of their partner.

Table 3 shows Cox proportional hazards models separately by age category and sex. Before 2005, being in a conjugal relationship was associated with a lower hazard of all-cause mortality for both females and males in all age groups compared to being not partnered. The protective association with being in a conjugal relationship was substantial with hazard rates for females 37–80% lower and hazard rates for males 38–66% lower. After 2005, the protective association with being in a conjugal relationship was substantially weaker but remained significant for females in all age groups, except those aged 20–29 years. Similarly, for men, being in a conjugal relationship remained significantly protective for males aged 40 years and older compared to being not partnered, but was no longer significant for men aged 30–39 years, and was associated with a significantly increased risk of mortality for males aged 20–29 years.

**Table 3:** Cox proportional hazards models for the effect of relationship status on all-cause mortality, by age and sex in the Africa Centre Demographic Information System, 2001–2011

Females										
	20–29 Years		30–39 Years		40–49 Years		50–59 Years		60 Years and over	
Number of deaths		1259		919		594		443		1260
	HR	95% CI	HR	95% CI	HR	95% CI	HR	95% CI	HR	95% CI
Relationship status before 2005 (ref: not in relationship)	1.00		1.00		1.00		1.00		1.00	
Non-conjugal relationship	0.71	(0.60 to 0.85)***	0.73	(0.60 to 0.87)**	0.88	(0.70 to 1.12)	1.07	(0.72 to 1.59)	1.50	(1.08 to 2.09)*
Conjugal relationship	0.30	(0.23 to 0.39)***	0.20	(0.16 to 0.26)***	0.28	(0.22 to 0.37)***	0.63	(0.47 to 0.86)**	0.51	(0.39 to 0.68)***
Relationship status after 2005 (ref: not in relationship)	1.00		1.00		1.00		1.00		1.00	
Non-conjugal relationship	1.34	(0.77 to 2.35)	1.39	(0.94 to 2.04)	1.04	(0.71 to 1.54)	1.31	(0.74 to 2.33)	1.09	(0.56 to 2.12)
Conjugal relationship	0.64	(0.35 to 1.18)	0.53	(0.35 to 0.79)**	0.45	(0.33 to 0.62)***	0.65	(0.49 to 0.87)**	0.73	(0.57 to 0.93)*

Males										
	20–29 years		30–39 years		40–49 years		50–59 years		60 years and over	
Number of deaths		1171		1139		858		581		897
	HR	95% CI	HR	95% CI	HR	95% CI	HR	95% CI	HR	95% CI
Relationship status before 2005 (ref: not in relationship)	1.00		1.00		1.00		1.00		1.00	
Non-conjugal relationship	0.87	(0.73 to 1.04)	0.66	(0.55 to 0.81)***	0.62	(0.48 to 0.80)***	0.86	(0.62 to 1.19)	1.37	(1.04 to 1.80)*
Conjugal relationship	0.62	(0.47 to 0.82)**	0.36	(0.29 to 0.45)***	0.34	(0.27 to 0.43)***	0.40	(0.30 to 0.54)***	0.59	(0.48 to 0.74)***
Relationship status after 2005 (ref: not in relationship)	1.00		1.00		1.00		1.00		1.00	
Non-conjugal relationship	2.10	(1.34 to 3.31)**	1.31	(0.82 to 2.09)	0.70	(0.46 to 1.06)	0.93	(0.53 to 1.62)	0.66	(0.32 to 1.34)
Conjugal relationship	1.83	(1.10 to 3.05)*	0.89	(0.56 to 1.42)	0.40	(0.28 to 0.57)***	0.50	(0.33 to 0.74)***	0.55	(0.41 to 0.75)***

Adjusted for age, age^2^ and education; *p<0.05, **p< 0.001, ***p<0.0001.

The association between being in a non-conjugal relationship and mortality is more complex. A significant protective effect of being in a non-conjugal relationship on the hazard of mortality before 2005 was observed in younger women, specifically those aged 20–29 and 30–39 years. In contrast, women aged 60 and older in a non-conjugal relationship had a significantly higher risk of mortality before 2005 than women of the same age who were not partnered. After 2005, being in a non-conjugal relationship was not associated with any significant change in the hazard of mortality compared to being not partnered.

For men, being in a non-conjugal relationship was associated with a significantly lower hazard of mortality before 2005 compared to being not partnered, among those aged 30–39 and 40–49 years only. After 2005, being in a non-conjugal relationship remained significantly protective compared to being not partnered only for those aged 40–49 years, and was associated with a significantly increased hazard of mortality for those aged 20–29 years.

Table 4 shows equivalent Cox models for non-AIDS deaths only. While precision in some age groups was reduced due to the smaller number of deaths, the HR estimates for men and women in conjugal and non-conjugal relationships were consistent with the size and pattern of estimates from the all-cause analysis, but not always their statistical significance. For men aged 20–29 years, the increased risk of non-AIDS mortality associated with being in a conjugal or non-conjugal relationship was attenuated and the estimates were no longer statistically significant.

**Table 4:** Cox proportional hazards models for the effect of relationship status on non-AIDs mortality, by age and sex in the Africa Centre Demographic Information System, 2001–2011

Females										
	20–29 Years		30–39 Years		40–49 Years		50–59 Years		60 Years and over	
Number of deaths		386		270		259		307		1195
	HR	95% CI	HR	95% CI	HR	95% CI	HR	95% CI	HR	95% CI
Relationship status before 2005 (ref: not in relationship)	1.00		1.00		1.00		1.00		1.00	
Non-conjugal relationship	0.70	(0.49 to 0.99)*	0.87	(0.59 to 1.27)	1.05	(0.68 to 1.60)	0.73	(0.40 to 1.32)	1.47	(1.03 to 2.10)
Conjugal relationship	0.44	(0.27 to 0.72)**	0.24	(0.15 to 0.40)***	0.34	(0.22 to 0.54)***	0.76	(0.53 to 1.09)	0.52	(0.39 to 0.70)***
Relationship status after 2005 (ref: not in relationship)	1.00		1.00		1.00		1.00		1.00	
Non-conjugal relationship	1.05	(0.49 to 2.28)	1.93	(1.00 to 3.73)*	1.03	(0.59 to 1.80)	1.07	(0.49 to 2.31)	1.03	(0.51 to 2.09)
Conjugal relationship	0.51	(0.22 to 1.18)	0.94	(0.49 to 1.81)	0.74	(0.50 to 1.09)	0.76	(0.55 to 1.06)	0.71	(0.55 to 0.91)**

Males										
	20–29 years		30–39 years		40–49 years		50–59 years		60 years and over	
Number of deaths		558		459		444		375		794
	HR	95% CI	HR	95% CI	HR	95% CI	HR	95% CI	HR	95% CI
Relationship status before 2005 (ref: not in relationship)	1.00		1.00		1.00		1.00		1.00	
Non-conjugal relationship	0.86	(0.66 to 1.12)	0.65	(0.47 to 0.91)*	0.52	(0.35 to 0.75)**	0.64	(0.41 to 1.01)	1.35	(1.00 to 1.81)*
Conjugal relationship	0.61	(0.40 to 0.94)*	0.38	(0.26 to 0.54)***	0.32	(0.23 to 0.44)***	0.39	(0.27 to 0.56)***	0.59	(0.47 to 0.75)***
Relationship status after 2005 (ref: not in relationship)	1.00		1.00		1.00		1.00		1.00	
Non-conjugal relationship	1.87	(1.04 to 3.37)*	0.79	(0.44 to 1.45)	0.42	(0.25 to 0.71)**	0.91	(0.46 to 1.77)	0.59	(0.27 to 1.32)
Conjugal relationship	1.43	(0.73 to 2.82)	0.60	(0.34 to 1.09)	0.30	(0.20 to 0.45)***	0.51	(0.32 to 0.83)**	0.56	(0.41 to 0.77)***

NB: Adjusted for age, age^2^ and education; *p<0.05, **p< 0.001, ***p<0.0001.

All models control for highest educational attainment, the inclusion of which made little substantive difference to the association between relationship status and mortality. Education was strongly related to mortality risk, with those who were least educated having a higher mortality risk as well as those whose educational attainment was unknown or missing. The models were also run excluding the missing category, but this did not substantively change the results.

It should be noted that we conducted a sensitivity analysis using different possible cut-off points in recognition that ART initiation and follow-up were rolled out from November 2004 to late 2007. The results did not change substantially; however, by using a cut-off point later than 2007, both changed the results and violated the proportional hazards assumption.

## Discussion

Studies conducted elsewhere have found that marriage has a protective effect on adult mortality.^2–4^ However, these studies were conducted in settings with lower levels of adult mortality than this study community in northern KwaZulu-Natal, where until the introduction of ART in 2005 the severe HIV epidemic led to very high levels of adult mortality.^11–13^ Given that there has been a well established and substantial decline in the proportion of married Zulu-speaking men and women,^14^ ^16^ we asserted that it was appropriate to examine differentials in mortality risks by types of relationships other than marital and non-marital. In the context of high levels of migration as well as low rates of marriage, we used population-based data collected prospectively, and categorised relationships by the extent of social connectedness based on whether or not the partners were members of the same household, and compared these individuals with those not partnered. Furthermore, we asserted that any mortality differentials by relationship status are likely to vary across the periods before and after treatment roll-out. This is the first investigation of the association between mortality and conjugal and non-conjugal relationships in an African population.

Consistent with other studies in Africa, dissolution rates increased with age.^27^ Relationship formation peaks in men at a later age than in women, with men being more likely to form new relationships at older ages than women of the same age.

Before 2005, individuals in conjugal relationships had a lower hazard of all-cause mortality in all age groups than not partnered men and women. In contrast, non-conjugal relationships were associated with a lower hazard of death in only a few age groups of women (20–29, 30–39 and 60 years and over) and men (30–39, 40–49 and 60 years and over). After ART introduction, the protective association of conjugal relationships was weaker but remained generally significant for men and women but not in non-conjugal relationships. In the later period, the association was reversed in young men (20–29 years) with mortality higher in conjugal and non-conjugal relationships compared with men not partnered. In a context where marriage rates are low and cohabitation is not universal, the findings suggest that conjugal relationships, with their higher degree of social connection through shared membership of the same household, affords men and women with greater protection against mortality.

The results of the analyses of non-AIDS mortality for men and women are similar to the pre-2005 and post-2005 estimates for all-cause mortality. The post-2005 estimates for young men (20–29 years) in conjugal and non-conjugal relationships were attenuated in the analysis of non-AIDS mortality but remained suggestive of an increased risk of mortality compared with those not partnered. Partnering in young men (20–29 years) is common. Post-2005, the proportion of young men in a conjugal relationship was small (15% of person-time), whereas about 70% of person-time was spent in a non-conjugal relationship. Union dissolution rates are higher for men in this age group compared with other age groups; however, we would not anticipate that instability in relationships would influence the estimates of mortality risk given the time-varying approach used in this analysis. Instead, we interpret this finding as suggesting that young men who have entered conjugal and non-conjugal relationships by this age have characteristics that differ more markedly from those not partnered than at older ages. Furthermore, these characteristics, for example, socioeconomic status (SES), may also be associated with increased mortality.^28^ ^29^ The increased risk of non-AIDS deaths in this age group of men is primarily due to accidental and intentional injuries.^30^

There are some limitations to our approach. It is possible that previous relationship status impacts mortality risk for a period after the status changes; however, we would expect that effect to diminish with time and have not incorporated lagged effects in our models. Given that the identity of the partner is not available in the ACDIS for those in a non-conjugal relationship, it was necessary to consider that a change in status from a non-conjugal to conjugal relationship was with the same partner, though this could occasionally be with a different partner. Similarly, on occasion, serial reports of a non-conjugal relationship could relate to different partners. Thus, our estimated formation and dissolution rates may underestimate the underlying rates in the population. In addition, we are only able to consider primary relationships because data regarding multiple partnerships and the number of lifetime partners for individuals or their current partners are not available. In the literature, it has been debated whether the concurrency behaviour of an individual's partners increases his or her own risk of HIV infection,^31–33^ while the number of lifetime partners is an established risk factor for HIV.^32^ ^34^ In this data set, the majority of partnerships cannot be linked, so we are unable to establish an individual's HIV risk due to their partner's concurrency; neither can we identify individuals in the population who have a higher risk of HIV and thus a potentially different mortality risk.^32^ ^35^ The way in which a relationship ends may be associated with differential mortality risk, but it was not possible to explore formal divorce separately given that few customary marriages end with a formal divorce. That said, the study also has a number of strengths: the inclusion of relationship status as a time-varying rather than a fixed covariate, prospective longitudinal data collection that minimises recall bias, and the availability of detailed information allowing relationships to be categorised as conjugal and non-conjugal.

There is much still to learn about the processes by which the timing and characteristics of a relationship impact on the health of men and women. Within our analysis, we controlled for educational attainment, which had a strong association with mortality risk, but we recognise that further work is needed to investigate socioeconomic gradients in relationship levels and patterns including age-specific formation, dissolution and duration. We anticipate that there may be socioeconomic differences in the pattern of conjugal relationships; however, these are likely to be complex given the declining marriage levels and rising age of first marriage. Appropriate measures of household-level and individual-level SES across time would be needed. Attributes of one’s partner may also determine the risk of marriage, risk of conjugal union and household SES, in addition to the risk of mortality. The effect of migration and cohabitation on mortality could also be explored and in doing so would allow for a better understanding of HIV and other health risks.

In conclusion, we have demonstrated that being in a conjugal relationship in this context is associated with lower all-cause mortality. This study contributes to the increasing body of evidence worldwide that family relationships and partnerships are important determinants of health and health behaviours^36^ and suggests that greater attention on couple-focused interventions informed by knowledge about couples’ social and residential arrangements may be an effective way of improving individual and couples’ joint health outcomes.

**What is already known on this subject**

- Multiple studies have shown that being married is associated with lower mortality among both men and women. However, previous studies have concentrated on higher income contexts and have not been conducted in settings where marriage rates are low and many couples live apart.

**What this study adds**

- The concepts of conjugal and non-conjugal relationships are used in place of the married/non-married dichotomy normally used. This study shows that in South Africa being in a conjugal relationship is associated with lower mortality than for those who were not partnered, for both men and women. In some age groups, being in a non-conjugal relationship was also associated with lower mortality, meaning that not being partnered was associated with the highest risk of death.
